# Spatio-temporal pattern evolution of China’s provincial tourism efficiency and development level based on DEA-MI model

**DOI:** 10.1038/s41598-023-46884-5

**Published:** 2023-11-18

**Authors:** Zhenjie Liao, Lijuan Zhang, Shan Liang

**Affiliations:** 1 School of Management, Guangzhou Huashang College, Guangzhou, 511300 China; 2 School of Economics, Guangzhou City University of Technology, Guangzhou, 510800 China

**Keywords:** Environmental social sciences, Mathematics and computing

## Abstract

The spatial differences of efficiency and development level of regional tourism are evident, and the dynamic grasp of their spatiotemporal evolution characteristics and coupling coordination relationship is important to promote high-quality sustainable tourism development. Herein, we measured the tourism development level of 31 provincial units in China during 2000–2020, introduced the data envelopment analysis-based Malmquist productivity index to determine tourism efficiency, used exploratory spatiotemporal data analysis methods to explore the spatiotemporal dynamic characteristics of regional tourism differences and spatial structure, constructed a coupled coordination model of tourism efficiency and development level and analyzed their coupling excellence and synergistic consistency. The results were as follows: (1) The spatial differences of comprehensive tourism efficiency from 31 provinces were evident, and the average situation showed high and low distribution characteristics in the east and west, respectively. The interannual changes showed a fluctuating downward trend, with the scale efficiency playing a supporting role for comprehensive efficiency and technical efficiency playing an influencing and restraining role. (2) Less fluctuation existed in the local spatial structure of tourism efficiency and development level; the direction of dependence was more stable, tourism development level was slightly more volatile, and the spatial dependency direction changes were similar. The tourism efficiency of local structure competition posture was stronger than that of collaboration, with the tourism development level collaboration integration being stronger. (3) The local spatial structure of tourism development level was more stable, the relative position of provincial units was more difficult to change, tourism efficiency of local spatial structure was unstable, and provincial units exhibited a greater possibility of change. (4) Tourism efficiency and the scale of the overall coupling degree and coupling coordination gradually improved, with similar spatiotemporal values. The overall coupling degree and coupling coordination degree of tourism efficiency and development level gradually improved, with similar spatiotemporal heterogeneity and volatility of local evolution. The area with a higher value coupling coordination degree spread slower and more widely.

## Introduction

The Chinese economy is undergoing a transition from rapid development to high-quality development, and optimizing the economic structure and transforming the mode of economic growth have become the main tasks at present. The high-quality development of the tourism industry is an important component of the spectrum of high-quality development. Since the reform and opening up, China's tourism industry has grown steadily. China's tourism industry has made tremendous achievements, becoming a major tourism country in the world and moving towards the goal of building a world tourism powerhouse. In 2019, the number of Chinese tourists exceeded 6 billion, and the total tourism revenue increased by 11% year-on-year, reaching 6.63 trillion yuan, which shows that China has achieved the leap from a big country of tourism resources to a big country of tourism economy, and is moving toward a strong tourism economy. The high-quality development of tourism will be an inevitable trend in line with the trend of the times. Under the new situation, both efficiency and development level are paid equal attention, and dynamically grasping the synergistic relationship between them has important theoretical and practical significance for high-quality tourism development. How to balance the efficiency and development level of tourism in a scientific and overall way, how to correctly grasp the relationship between breaking old momentum and cultivating new momentum, self-development and coordinated development, is the key to the high-quality development of China's tourism. With the rapid growth of the tourism industry, the single factor tourism resource expansion model of "high investment, low output, and low efficiency" has become a characteristic of the development of the tourism industry in multiple regions across the country. At the same time, tourism development units at different spatial scales tend to diversify, and the tourism regional system within the scope of cities, provinces, urban agglomerations, etc. serves as an important carrier for tourism development, providing it with broad extension space. The quality and development mode of tourism economic growth have become a hot topic of concern for scholars at home and abroad. However, the quality of tourism economic growth covers multiple aspects, and accurately measuring and objectively evaluating the quality of tourism economic growth is a key issue that urgently needs to be addressed. Scholars have attempted to construct a multi-dimensional and multi connotation evaluation index system to comprehensively evaluate the quality of tourism economic growth. Although different research conclusions have been drawn, there is a certain consensus on the importance of economic efficiency to the quality of economic growth to some extent. On the one hand, even though the focus on evaluating the quality of tourism economic growth varies, tourism economic efficiency is regarded as one of the important or core indicators; On the other hand, from a narrow perspective, the quality of economic growth can be measured by economic efficiency. Tourism efficiency, as an important measure of the ability of tourism economic entities to utilize resources and maximize total surplus for all stakeholders, can be used to more intuitively evaluate the rationality of the input and output of the tourism industry. However, existing research on tourism efficiency focuses more on the comprehensive output efficiency of multiple factor inputs, and it is difficult to quantify the output efficiency of a single factor, resulting in the factor transformation efficiency of the tourism industry still maintaining a "black box" state, making it difficult to propose targeted policy recommendations to promote high-quality development of the tourism economy in practice.

Efficiency refers to the evaluation method of using resources most effectively to meet the set wishes and needs under given conditions such as investment and technology^1^. Tourism efficiency refers to the economic benefits that a region can achieve after applying a certain cost. Tourism efficiency reflects the internal connection and ratio relationship between the input and output of tourism economic activities. Tourism efficiency affects the competitiveness and sustainable development of destinations, and also promotes the transformation and upgrading of the tourism industry^2^. Since the reform and opening up, the scale of China's tourism industry has continuously expanded and gradually developed into a strategic pillar industry of the national economy. A good tourism industrial structure can effectively guide factor input, improve input–output conversion efficiency, accelerate the flow of new and old kinetic energy, ensure the continuous and stable operation of variables represented by technological progress, fully leverage its spillover effects, and achieve economies of scale. The adjustment of tourism development level and the allocation of technological elements need to be coordinated to maximize the improvement of tourism efficiency, and achieve the optimal energy efficiency of the tourism development quality control system. Tourism efficiency is a comprehensive indicator that reflects the utilization level of tourism development resources and the sustainable development ability of tourism. Improving tourism efficiency plays an important role in promoting the transformation and upgrading of the tourism economy and ensuring the sustainable and healthy development of the tourism industry. The degree of coordinated development of regional tourism efficiency plays a promoting role in the sustainable economic development ability and economic benefits of tourism destinations, while also reflecting the efficient operation of tourism economic activities in the region^3,4^. At present, scholars' research trends on tourism efficiency are mainly reflected in the following aspects. (1) The research content has shifted from single tourism efficiency evaluations such as management efficiency^5^, operational efficiency^6^, and tourism transportation efficiency^7^ for the tourism industry to comprehensive efficiency evaluations such as tourism ecological efficiency^8^, tourism poverty alleviation efficiency^9^, and regional tourism efficiency^10^; (2) The research method has gradually shifted from qualitative evaluation of tourism efficiency to comprehensive quantitative analysis using multiple models such as data envelopment analysis^11^, SBM Malmquist model^12^, DEA-SNA model^13^, etc.; (3) The research area has transformed from large-scale regions such as national level^14^, provincial level^15^, and Yangtze River Delta^16^ to medium-sized regions such as national level scenic spots^17^ and A-level tourist attractions^18^; (4) The depth of research has shifted from exploring the spatiotemporal variation characteristics of tourism efficiency^19^ to exploring the driving mechanisms^20^ of its spatiotemporal differentiation from factors such as natural environment^21,22^, economic development level^23,24^, tourism resource endowment^25,26^, transportation conditions^27,28^, and human institutional supply^29^.

In general, studies related to regional tourism differences and tourism spatial structure are more comprehensive, although some limitations remain. efficiency and development level are given equal importance in the era of high-quality tourism development; however, there are few comprehensive measures of tourism quality based on tourism efficiency and development level. Classical mathematical statistics and exploratory spatial data analyses primarily measure cross-sectional characteristics of spatial association and interaction mechanisms^30^. Overall, studies that ignore the spatial dimension and static studies that ignore the temporal dimension show limited regional spatial and temporal differences. The theory of time and space emphasizes the ability to understand and analyze things from these perspectives. A spatiotemporal perspective is crucial to explore regional tourism development. A tourism region is a composite geographic system that gathers multiple functions constantly adjusting, and should combine temporal and spatial attributes when exploring its regional differences and spatial structure to more comprehensively reveal the dynamic divergence law of regional tourism development. China's tourism industry has accelerated the expansion of its market size after over 30 years of development and has become an important industry in the national economy in terms of time dimension. Overall, tourism intensification has increased every year, tourism efficiency has been optimized every year, and the development trend of the tourism industry is good. The eastern region has more advantages in terms of geographical location, economic strength, infrastructure construction, and so on, with a good tourism development atmosphere and a high starting point. The progress space for tourism efficiency is relatively small and the speed is relatively slow. In comparison, the foundation of tourism development in the western region is weak, and the advantages of tourism latecomers are gradually becoming prominent. The tourism development model is gradually shifting from extensive to intensive, and tourism efficiency is rapidly improving. The local spatial structure is highly dynamic.

This article refers to previous research results^4^, explored the development of regional tourism industry from the perspective of time and space. The main objectives were to: (1) measure the tourism development level of 31 provincial units in China from 2000 to 2020, (2) determine and decompose tourism efficiency using the data envelopment analysis (DEA-BCC) model and Malmquist productivity index, and (3) explore the dynamic evolution of local tourism’s spatial structure using local indicators of spatial association (LISA) time path and LISA spatiotemporal leap based on the exploratory spatiotemporal data analysis (ESTDA) framework proposed by Rey et al.^31^. The coupled coordination degree model of tourism efficiency and development level was constructed to explore the coupled coordination relationship between them. This study provides a new research perspective for in-depth coupling of the spatiotemporal evolution of regional tourism and offers a scientific basis to improve the quality, efficiency, and synergistic sustainable development of regional tourism.

## Materials and methods

### Research methods

#### DEA-BCC model

Data envelopment analysis is a linear programming method based on the measurement of the efficiency frontier under the input–output comparison of multiple decision-making units. The DEA-BCC model breaks the assumption of constant payoff of scale in the Charnes–Cooper–Rhodes model; it further decomposes the static combined efficiency of the decision-making units under variable payoff of scale into pure technical efficiency and development level efficiency^32^ and uses the output-oriented BBC model to measure tourism efficiency and analyze the current situation of tourism factor utilization.

#### Malmquist productivity index model

The DEA-based Malmquist productivity index decomposes total factor productivity of tourism to reflect the temporal trends of tourism efficiency and the main factors leading to the generation of changes. It is expressed as^33^:

1 $$M_{0} (x_{t} ,y_{t} ,x_{t + 1} ,y_{t + 1} ) = \sqrt {\frac{{D_{0}^{t} (x_{t + 1,} y_{t + 1} )}}{{D_{0}^{t} (x_{t,} y_{t} )}} \times \frac{{D_{0}^{t + 1} (x_{t + 1,} y_{t + 1} )}}{{D_{0}^{t + 1} (x_{t,} y_{t} )}}}$$

2 $$M_{0} (x_{t} ,y_{t} ,x_{t + 1} ,y_{t + 1} ) = \frac{{D_{0}^{t + 1} (x_{t + 1,} y_{t + 1} )}}{{D_{0}^{t + 1} (x_{t,} y_{t} )}} \times \sqrt {\frac{{D_{0}^{t} (x_{t + 1,} y_{t + 1} )}}{{D_{0}^{t + 1} (x_{t + 1,} y_{t + 1} )}} \times \frac{{D_{0}^{t} (x_{t,} y_{t} )}}{{D_{0}^{t + 1} (x_{t,} y_{t} )}}}$$

where xi and xt + 1 are the input vectors of t and t + 1 respectively; yi and yt + 1 are the output vectors of t period and t + 1 period respectively; $D_{0}^{t} (x_{t} ,y_{t} )$ and $D_{0}^{t} (x_{t + 1} ,y_{t + 1} )$ are the distance functions of the decision-making units of the period t and the period t + 1 with the reference to the technological frontier of the period t; $D_{0}^{t + 1} (x_{t} ,y_{t} )$ and $D_{0}^{t + 1} (x_{t + 1} ,y_{t + 1} )$ are the distance functions of the decision-making units of the period t and the period t + 1 with the reference to the technological frontier of the period t + 1; $M_{0} (x_{t} ,y_{t} ,x_{t + 1} ,y_{t + 1} )$ refers to the total factor productivity index (TFPCH). A value greater than 1 implies that the total factor productivity is increased. A value less than 1 implies that the total factor productivity is decreased. A value equal to 1 indicates that the total factor productivity is unchanged. The first item on the right side of Eq. (2) represents the technical efficiency change (EFFCH) from t to t + 1, and the second item represents the technical progress change (TECH).

Among them, the change of technical efficiency can be divided into the change of scale efficiency (SECH) and the change of pure technical efficiency (PECH). Therefore, formula (1) can be further decomposed into:

3 $$M_{0} (x_{t} ,y_{t} ,x_{t + 1} ,y_{t + 1} ) = \frac{{S_{0}^{t} (x_{t} ,y_{t} )}}{{S_{0}^{t + 1} (x_{t + 1} ,y_{t + 1} )}} \times \frac{{D_{0}^{t} (x_{t + 1} ,y_{t + 1} /VRS)}}{{D_{0}^{t} (x_{t} ,y_{t} /VRS)}} \times \sqrt {\frac{{D_{0}^{t} (x_{t + 1} ,y_{t + 1} )}}{{D_{0}^{t + 1} (x_{t + 1} ,y_{t + 1} )}} \times \frac{{D_{0}^{t} (x_{t} ,y_{t} )}}{{D_{0}^{t + 1} (x_{t} ,y_{t} )}}}$$

where VRS represents the change of return to scale; CRS indicates that the return to scale remains unchanged;$S_{0}^{t} (x_{t} ,y_{t} )$ is the scale function of the period t with the technology frontier of the period t as the reference; $S_{0}^{t} (x_{t + 1} ,y_{t + 1} )$ is the scale function of the t + 1 period with the technology frontier of the t + 1 period as the reference. The first item on the right side of the equation represents the change in scale efficiency (SECH) from t to t + 1, and the second item represents the change in pure technical efficiency (PECH).

#### Coupling coordination degree model

Coupling degree is an index to quantitatively measure the degree of mutual influence and interaction between two or more systems. The coupling degree model of tourism efficiency and development level is constructed by referring to relevant research results combined with the actual research using the following formula^34^:

4 $$C = \left\{ {f(x)g(y)/[(f(x) + g(y))/2]^{2} } \right.^{k}$$

where C is the coupling degree of tourism efficiency and development level, 0 < C < 1. A larger C value corresponds to better coupling; f(x) and g(x) are the tourism efficiency index and tourism development level index, respectively; k is the adjustment coefficient (it is generally 2 ≤ k ≤ 5). The k value in this paper was taken as 2 since the coupling degree model consisted of two subsystems.

The coupling coordination model was used to further explore the excellence of coupling between tourism efficiency and development level, together with the consistency characteristics of the synergistic effect, and their overall efficacies. Its calculation is based on the following formula^35^:

5 $$D = \sqrt {C \times T} ,\;T = {\upalpha }f(x) + {\upbeta }g(y)$$

where D is the coupling coordination degree of tourism efficiency and tourism development level; T is the comprehensive coordination index of both; α and β are coefficients to be determined, and α + β = 1. On the basis of previous studies^4^, this article believes that the two subsystems of tourism efficiency and tourism development level are equally important; Therefore, let α = β = 0.5.

### Indicator Selection and Data Sources

Tourism efficiency mainly depends on input and output indicators. Input indicators involving the most basic factors of production in classical economics mainly include land, labor, and capital^36^. Due to the difficulty in obtaining provincial tourism land data, most relevant studies have not included it in the input variable indicators^37^. Tourism employees are the most direct providers of tourism services, and their numbers are the most ideal measure of the labor factor. However, affected by the comprehensive characteristics of the industry, most provinces lack statistics on this indicator. Therefore, the labor factor indicator is replaced with the number of employees in the tertiary industry. This indicator has strong data availability and almost covers all direct and indirect employment related to the tourism industry. This amplifies the scale of the input of the labor factor; however, it considers the comprehensive nature of the tourism industry to a certain extent. The capital factor is an important support for tourism activities, and most provinces lack official statistics on fixed investment in tourism; therefore, the number of 3A (or three-star) grade and above tourist attractions (points), star-rated hotels, and travel agencies reflecting the status of tourism resources and tourism services are used as alternative input indicators for the capital element of tourism. Meanwhile, the total number of tourist arrivals and total tourism revenue are selected as the primary indicators of the direct output of tourism activities. Herein, total tourism headcount and total tourism revenue indicators were selected to construct the tourism development level measurement model to maintain data consistency and comparability of the results. The tourism development level measurement model is^18^:

6 $$SP_{n} = \sum\limits_{i = 1}^{n} {P_{i} S_{i} }$$

where Pi is the weight calculated by applying the entropy value method and Si is the dimensionless value of indicator i. Meanwhile, the total tourism revenue is deflated by using the Consumer Price Index (CPI) of each year as the base period in 2000 to eliminate the influence of price fluctuations. Simultaneously, the total tourism income was deflated using the consumer price index of each year compared with the base period in 2000 to eliminate the influence of price fluctuations.

**Figure 1:**
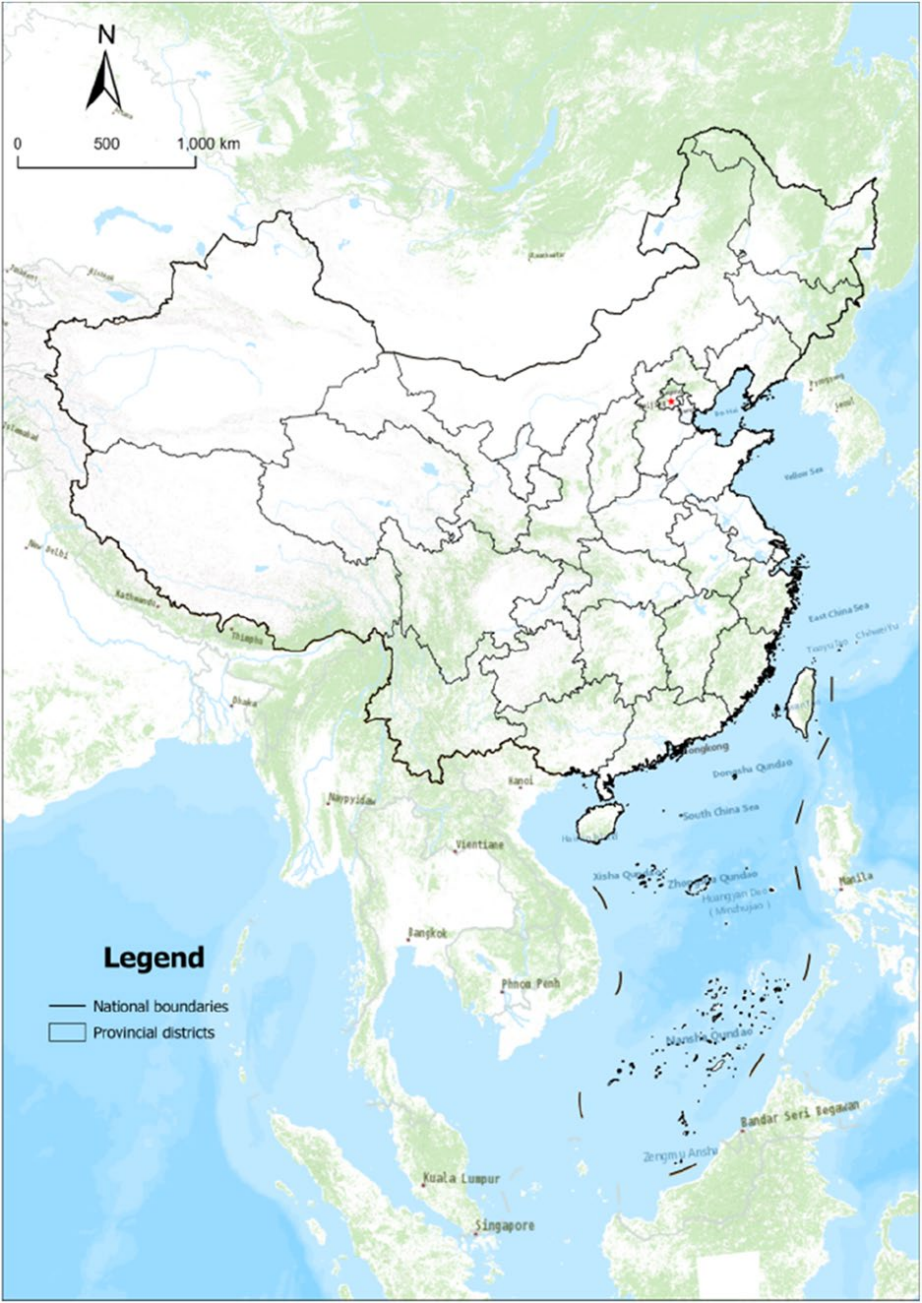
Study area.

The administrative boundary vector data of 31 provincial units in China was extracted based on the 1:7,000,000 China administrative division map of the National Bureau of Surveying, Mapping, and Geographic Information (Fig. 1). The data in this study were primarily obtained from the China Regional Economic Statistical Yearbook 2000–2020 and the 2000–2020 provincial (city) statistical yearbooks, tourism yearbooks, and national economic and social development statistical bulletins. Some missing index data were calculated and supplemented using index smoothing.

## General characteristics of provincial tourism efficiency and tourism development level in China

### Static characteristics of tourism efficiency

**Figure 2:**
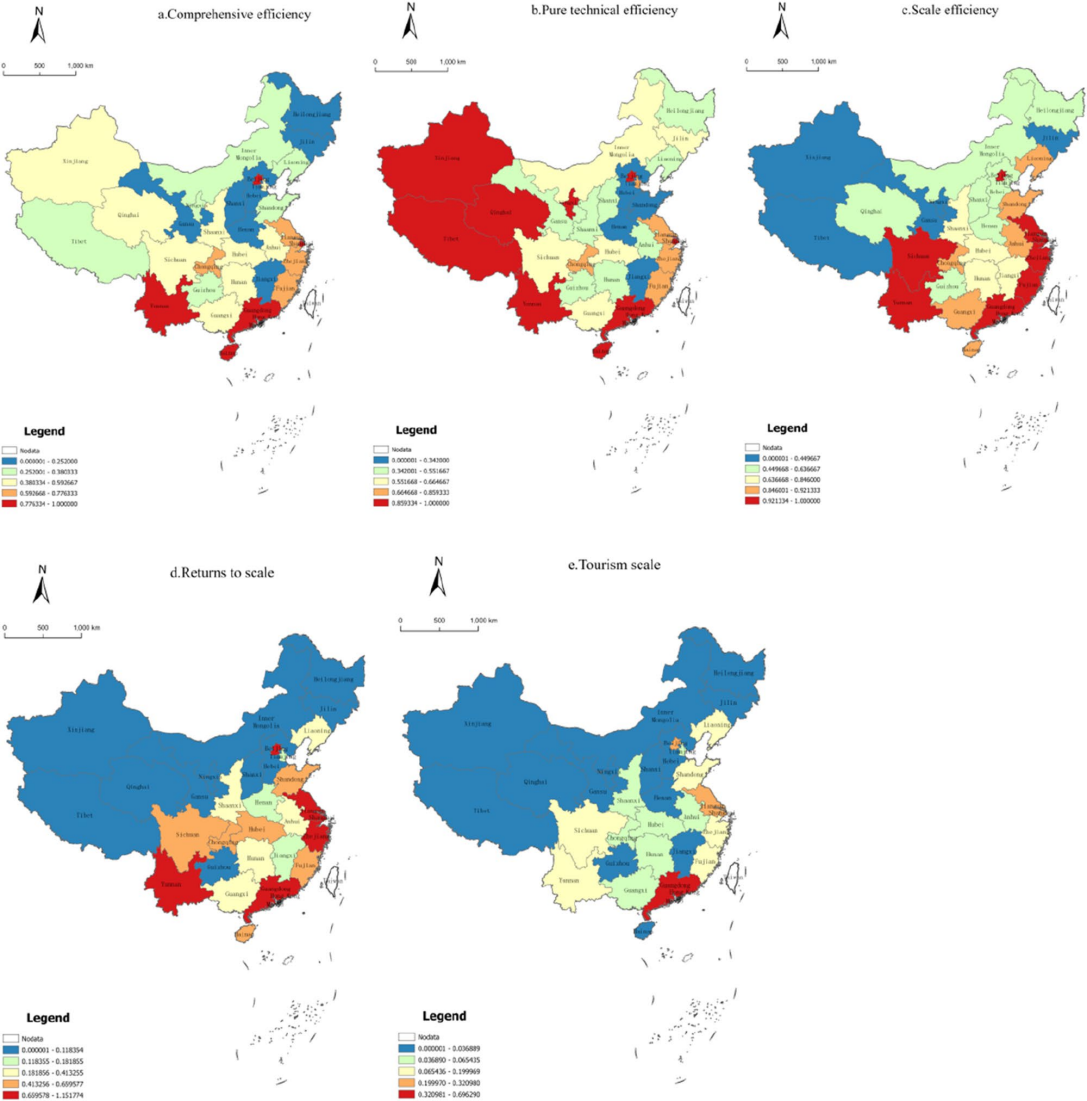
Average performance of tourism static efficiency and tourism development level in China from 2000 to 2020.

The DEA-BCC model was used to measure the tourism efficiency of 31 provincial units in China from 2000 to 2020, and the average values of the overall tourism efficiency, pure technical efficiency, and scale efficiency of each provincial unit were calculated separately. The Nature Breaks method of ArcGIS 10.2 software was used to classify the three types of efficiency. The average measurement results were classified into five levels: low efficiency, medium–low efficiency, medium efficiency, medium–high efficiency, and high efficiency (Fig. 2).

The critical values of all levels of comprehensive efficiency were 0.186, 0.381, 0.593, and 0.776, respectively (Fig. 2a), and the average value of comprehensive efficiency was 0.466. This reached 46.6% of the ideal state, which lied in the medium efficiency level and could be further improved. No province exhibited a static integrated efficiency mean value on the production frontier. The overall average comprehensive efficiency of Chinese provincial tourism showed high and low distribution characteristics in the east and west, respectively.

The critical values of pure technical efficiency at all levels were 0.342, 0.552, 0.665, and 0.859, respectively (average = 0.605; Fig. 2b). This average lay in the medium efficiency level and could be further improved. On average, the high spatial distribution in the east and low spatial distribution in the west is highly similar to that observed in the comprehensive efficiency analysis. This indicated that the organization and management, institutional arrangement, and technical level of tourism development (such as organization and management, institutional arrangement, and innovation ability) are not mature enough and exhibit a strong inhibiting effect on the overall tourism efficiency. Our results indicated that Guangdong Province, Beijing, and Yunnan Province reached the frontier side of pure technical efficiency, with better adeptness and promotion of technology.

The critical scale efficiency values at all levels were 0.118, 0.413, 0.659, and 0.735, respectively (average = 0.482; Fig. 2c). This average lay in the medium–high efficiency level, and the average value was significantly larger than that of technical efficiency. This plays a supporting role for comprehensive efficiency, whereas pure technical efficiency plays an influencing and restraining role. Therefore, tourism efficiency should be improved to improve technical efficiency in the studied 31 provinces.

In 2020, the number of provinces with decreasing, constant, and increasing returns to scale was 15, 11, and 5, respectively (Fig. 2d). Some provinces (35.48%) exhibited constant returns to scale, and the tourism factor inputs and outputs were optimal. Meanwhile, 48.39% of the provinces exhibited decreasing returns to scale with inefficient use of tourism resources and redundancy of factor inputs, and the scale of inputs could be reduced within a certain range. A smaller fraction of provinces (16.13%) exhibited increasing scale payoffs that are expected to obtain greater returns by continuing to expand tourism factor inputs.

### Dynamic Characteristics of China's Provincial Tourism Efficiency

The Malmquist index model was used to analyze the specific impact of technical efficiency and technological progress on total factor productivity and further analyze the dynamic change process of China's provincial tourism efficiency (Table 1).

**Table 1:** The Malmquist production index and its decomposition of tourism efficiency in China from 2000 to 2020.

Time period	EFFCH	TECHCH	PECH	SECH	TFPCH
2000–2001	0.6294	0.6209	0.6309	0.6898	0.6810
2001–2002	0.6745	0.6387	0.6035	0.6710	0.6049
2002–2003	0.6221	0.6598	0.6156	0.6101	0.6767
2003–2004	0.6344	0.7919	0.7317	0.6486	0.6283
2004–2005	0.7062	0.6388	0.7165	0.6362	0.7895
2005–2006	0.7692	0.7875	0.7403	0.7737	0.7915
2006–2007	0.7429	0.7172	0.7106	0.8556	0.8307
2007–2008	0.8486	0.8497	0.7809	0.8358	0.8998
2008–2009	0.8923	0.8340	0.7384	0.7761	0.7311
2009–2010	0.8303	0.8426	0.8389	0.7009	0.7512
2010–2011	0.8934	0.8871	0.7416	0.8158	0.7871
2011–2012	0.9987	0.9090	0.9497	0.9393	0.8457
2012–2013	0.8137	0.8883	0.8202	0.8241	0.8089
2013–2014	0.9951	0.8692	0.8506	0.8448	0.8387
2014–2015	0.9915	0.9175	0.9368	0.8179	0.9180
2015–2016	1.0641	1.0790	1.0459	1.0572	1.0643
2016–2017	1.0554	1.0483	1.0302	1.0840	1.0427
2017–2018	1.0434	1.0788	1.0108	1.0032	1.0755
2018–2019	1.0595	1.0717	1.0140	1.0496	1.0649
2019–2020	0.8387	0.8511	0.8024	0.8739	0.8035
Average value	0.8551	0.8491	0.8155	0.8254	0.8317

*EFFCH* technical efficiency change, *PECH* pure technical efficiency, *SECH* change of scale efficiency, *TECH* technical progress change, *TFPCH* total factor productivity index.

## Spatial and temporal dynamic evolutionary characteristics of tourism efficiency and tourism development level in Chinese provinces

### LISA time path analysis

**Figure 3:**
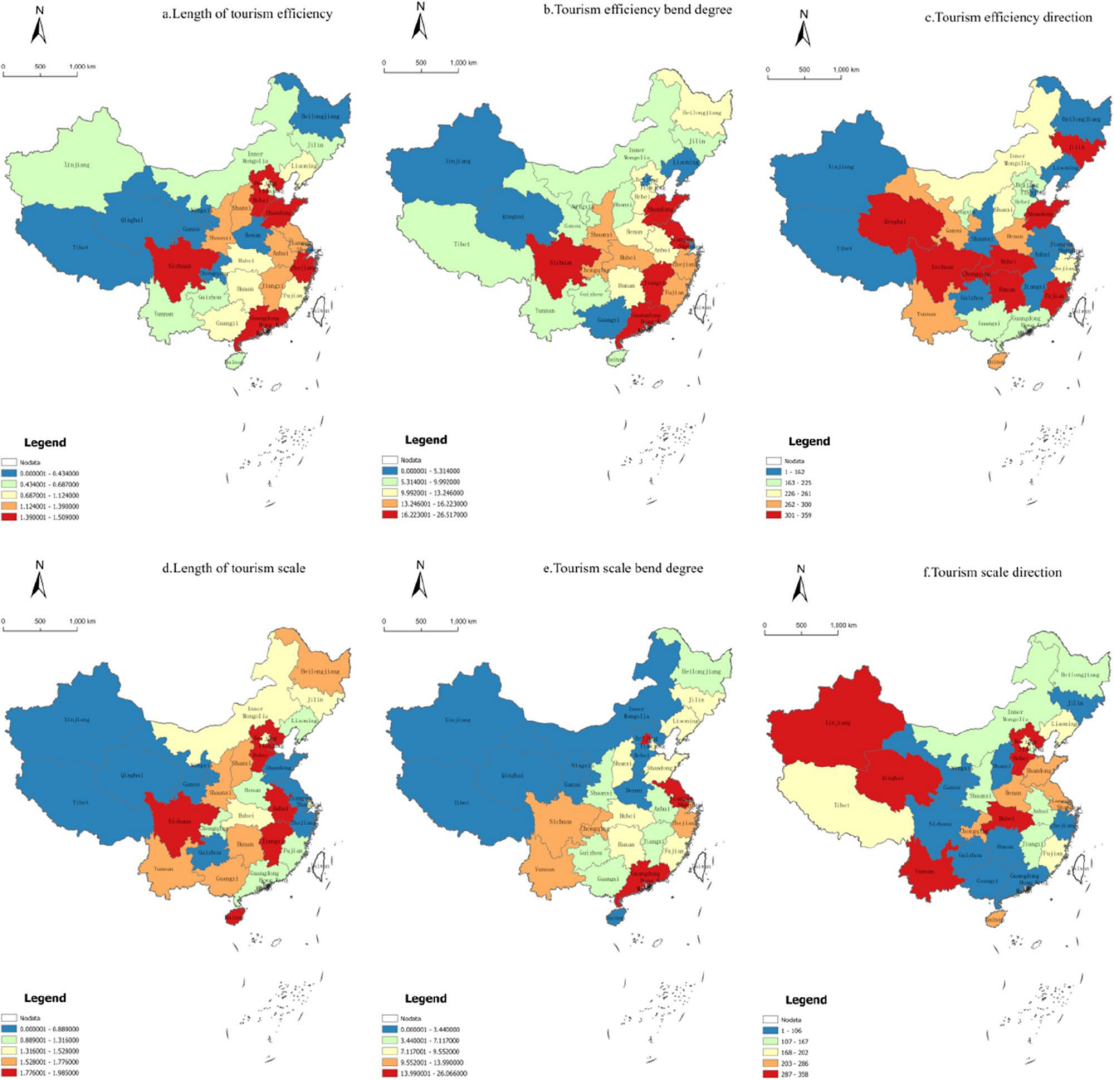
Spatial distribution of LISA time paths for tourism efficiency and tourism development level in China.

The length, curvature, and direction of the LISA time path of each provincial unit were calculated by simulating the specific positions of 31 Chinese provinces in the Moran scatter diagram of tourism efficiency and development level in 2000, 2010, and 2020. The spatiotemporal dynamic characteristics of local spatial structure dynamics, spatially dependent directional fluctuation, and spatial integration of tourism efficiency and development level were analyzed in Chinese provinces. The natural breakpoint method was used to classify the length and curvature of the LISA time path of China's provincial tourism efficiency and development level into four classes using ArcGIS 10.2 software, and the transfer direction of LISA coordinates were calculated for each province (Fig. 3).

#### LISA time path length

The mean value of tourism efficiency LISA time path length was 0.871 (Fig. 3a). Sixteen provinces were below the mean value; this accounted for 51.61% of the total. The local spatial structure was relatively stable, with the highest LISA time path length value of 1.509 in Guangdong Province and the lowest value of 0.225 in Henan Province. The extreme path length difference was small, and the local spatial structure barely fluctuated. The LISA time path length values of Chinese provinces gradually increased from the eastern region to the western region between 2000 and 2020. This indicated that the spatial structure of tourism efficiency was more stable in the eastern region than in the western region. This was mainly because the eastern region exhibits more advantages in terms of geographical location, economic strength, and infrastructure construction. It offers a good starting point for tourism development but less room for the progress of tourism efficiency. In contrast, the western region has a weak foundation for tourism development, and the later advantages of tourism are gradually highlighted. Here, the tourism development mode gradually changes from rough and loose to intensive, with tourism efficiency exhibiting faster improvement and the local spatial structure being more dynamic than that in the eastern region.

The mean value of the tourism development level LISA time path length was 1.317, and the number of provinces below the mean value was 14 (Fig. 3d); this accounted for 45.16% of the overall provinces. This shows that the local spatial structure of the tourism development level was more stable and less volatile than tourism efficiency. A total of 10 provinces exhibited LISA time path lengths greater than 1.5: Hubei province (1.5.28), Yunnan province (1.574), Shanxi province (1.611), Shaanxi province (1.692), Hunan province (1.695), Guangxi province (1.715), Heilongjiang province (1.776), Sichuan province (1.826), Hainan province (1.912), and Guangdong province (2.012). The shortest path length of Zhejiang Province was 0.455, and the extreme path length difference was large. Rapid economic growth has strongly driven the development of tourism development level in provinces with a good tourism development base. Meanwhile, tourism with an excellent development momentum and a highly fluctuating local spatial structure of tourism development level has emerged as a new economic growth point. The Yangtze River Delta city cluster, Pearl River Delta city cluster, and Beijing-Tianjin-Hebei city cluster for the path length of the three high-value areas. The core city tourism development level pole nucleus radiation role was brought into play, the effectiveness of regional tourism collaboration gradually emerged, there was a fast tourism development level growth rate, and there was a larger degree of fluctuation.

#### LISA time path direction

The direction of movement of the LISA coordinate points of each provincial unit was divided into four categories: (1) the 0°–90° direction was a win–win situation, wherein the provincial unit and its neighboring units showed positive synergistic growth compared with the average, the same below; (2) the 90°–180° direction was a lose-win situation, where the provincial unit and its neighboring units showed a reverse growth direction. The provincial unit itself exhibited low growth and the neighboring units exhibited high growth; (3) the 180°–270° direction was a lose-lose situation, and the provincial unit and its neighboring provinces exhibited negative synergistic growth; (4) the 270°–360° direction was a win-lose situation, the provincial unit and its neighboring provinces exhibited a reversed growth direction. The provincial unit exhibited high growth and the neighboring units exhibited low growth.

Tourism efficiency direction reversed the growth of 11 provinces. This accounted for 35.48% of the overall tourism efficiency local structure and showed that competitive dynamics were stronger than collaborative dynamics (Fig. 3c). There were 8 provinces with win-lose dynamics, 7 provinces with lose-win dynamics, and 4 provinces with win–win dynamics. Tourism development level direction showed win–win dynamics in 6 provinces, win-lose dynamics in 7 provinces, lose-win dynamics in 11 provinces, and reversed growth in 7 provinces (Fig. 3f). The tourism development level presented synergistic, low growth characteristics. Regional tourism collaboration methods provide the means for transformation and improvement, and a new common growth point of tourism development level is required.

### LISA spatiotemporal leap analysis

The spatiotemporal leap analysis method was used to further describe the local spatial correlation type of LISA coordinate points and the process of Moran scatter plot evolution among different local types (Table 2).

**Table 2:** Transition probability matrix of Local Moran's I.

Travel Efficiency					tourism development level			
	HHt + 1	HLt + 1	LHt + 1	LLt + 1	HHt + 1	HLt + 1	LHt + 1	LLt + 1
HHt	I(3,14.29)	III(5,23.8)	II(6,28.6)	IV(0,0)	I(2,9.52)	III(12,37.56)	II(5,18.69)	IV(0,0)
HLt	II(0,0)	I(0,0)	III(0,0)	IV(9,42.86)	II(3,21.27)	I(6,0.65)	III((5,11.32)	IV(0,0)
LHt	IV(7,0.36)	I(12,35.11)	III(3,10.21)	IV(0,0)	IV(0,0)	I(8,21.6)	III(0,0)	IV(0,0)
LLt	I(0,0)	II(0,0)	IV(0,0)	III(9,0.69)	I(18,58.27)	II(0,0)	IV(11,25.46)	III(0,0)

There were 13 provinces wherein tourism efficiency had undergone a spatiotemporal leap; this accounted for 49.35% of the total. The transfer of Local Moran's I between types was more active, the local regional structure of tourism efficiency was unstable, and it was easier for provincial units to change their relative positions. There were eight type IV provinces lacking a spatiotemporal leap; this accounted for 25.81% of the total. Therefore, the tourism efficiency of provincial units still had a certain transfer inertia. There were 10 type III provinces with a synergistic leap; this accounted for 32.26% of the total number of leaping provinces. The local spatial structure of provincial unit tourism efficiency was more influenced by its own factors and less influenced by the spillover of neighboring units. The number of HH type provinces increased from three to seven, and the overall agglomeration of provinces increased with higher tourism efficiency.

The local spatial structure of China's provincial tourism efficiency remained unstable. There was a certain transfer inertia of provincial units; however, a greater possibility of change existed. The local spatial structure of the tourism development level was more stable, and it was more difficult to change the relative position of provincial units. It was feasible to enhance tourism efficiency by adjusting the scale of tourism; however, there was a limited effect from the point of view of the ease of transfer of provincial units. Therefore, there should be a focus on the configuration and regulation of technical factors to optimize the energy efficiency of the system of quality regulation of tourism development. The local spatial structure of tourism efficiency and tourism development level will be influenced by neighboring units from the viewpoint of provincial unit transfer subjectivity or independence. However, it was still influenced by their own factors; therefore, Chinese provinces should carry out tourism collaboration and competition under the premise of improving the quality of their own tourism development, pay attention to maintain the subjectivity and independence of their own tourism economy, improve the subjective initiative of tourism development, combine their own location conditions, resource endowment, and economic foundation based on the development orientation and direction of urban clusters and economic zones. This should help find the key direction of dislocation development, avoid the problem of homogeneous development, and achieve differentiated synergistic development.

## Coupled coordination relationship between tourism efficiency and tourism development level in Chinese provinces

**Figure 4:**
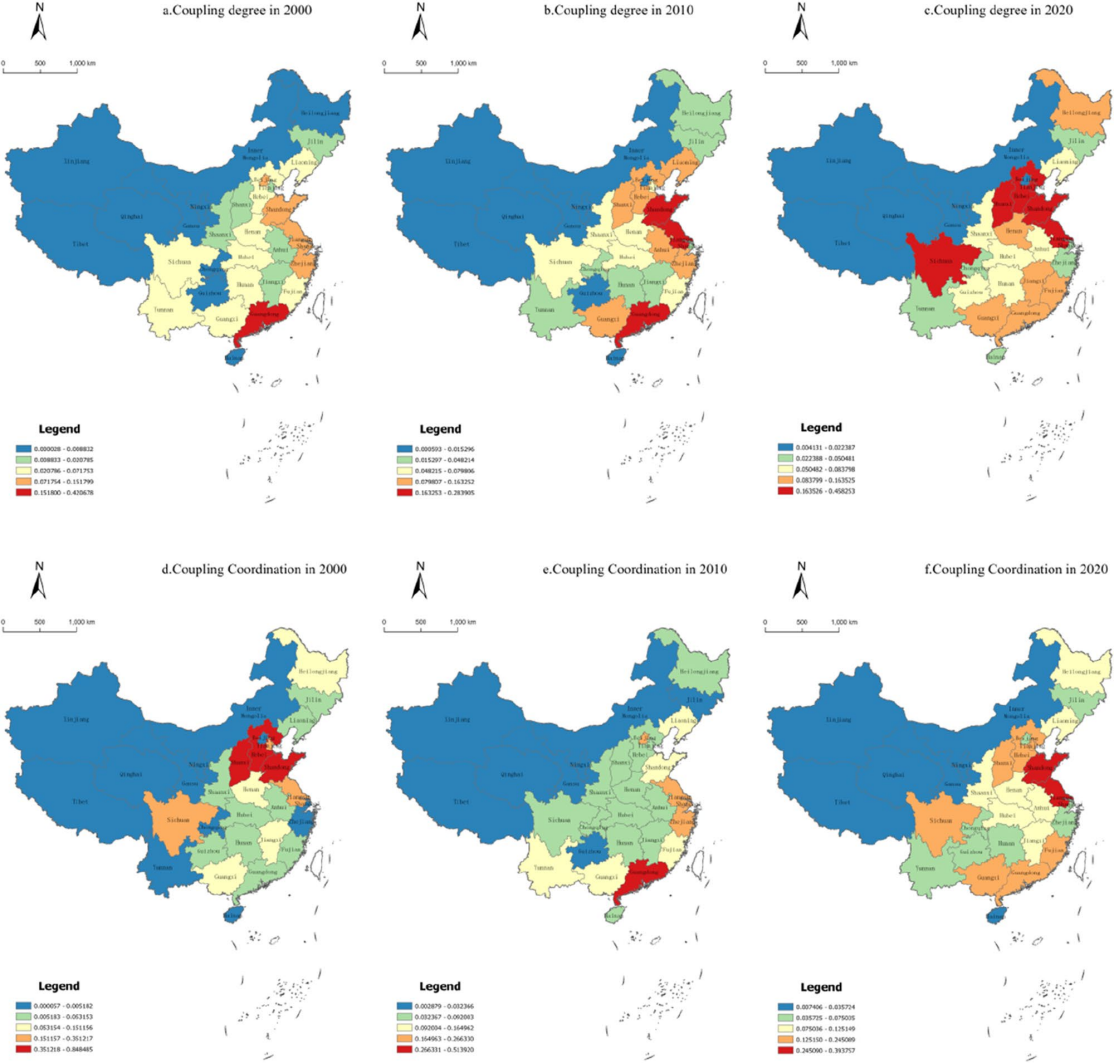
Spatial distribution of the coupling degree and coordination coupling degree of tourism efficiency and tourism development level in China.

The coupling degree model of tourism efficiency and tourism development level from 2000 to 2020 was constructed using Eq. (6) with reference to relevant research results^38–40^. The coupling degree was divided into four types: (1) When 0 ≤ C ≤ 0.03 is a low coupling period, there is a game between tourism efficiency and tourism development level. When C = 0, the two are in an unrelated state and develop towards disorder; (2) During the antagonistic period of 0.03 < C ≤ 0.05, the interaction between tourism efficiency and tourism development level strengthens, leading to the phenomenon of occupying the other party's development space; (3) 0.05 < C ≤ 0.15 is the period of adjustment, where tourism efficiency and tourism development level begin to balance and cooperate with each other, showing a benign coupling characteristic; (4)0.15 < C ≤ 1.0 is the period of coordinated coupling, and the benign coupling between tourism efficiency and tourism development level is becoming stronger and gradually developing towards an orderly direction. When C = 1.0, the two achieve benign resonance coupling and tend towards a new ordered structure. ArcGIS 10.2 software was used to plot the spatial distribution of the coupling degree in 2000, 2010, and 2020 (Fig. 4).

The mean coupling degree of provincial tourism efficiency and development level in China was 0.051, 0.071, and 0.111 in 2000, 2010, and 2020, respectively. The highest mean coupling degree was 0.632 in 2019. The correlation between them gradually increased, and the overall level of coupling degree showed an upward trend. The distribution of high value points of the coupling degree was scattered, and only Guangdong Province (0.421) was in the coordinated coupling period. Nine provinces were in the grinding period, and the remaining provinces were in the low coupling period. The coupling degree of 10 provinces was below 0.001, tourism efficiency and development level were nearly irrelevant, and the regional differences were significant. 2010 remained in the low coupling period as a whole, but the coupling performance was better than that in 2000. The number of provinces in the coordinated coupling and grinding period were three and nine, accounting for 9.67% and 29.03% of the total, respectively. Overall, there was an antagonistic period in 2020. The coupling degree in the Beijing–Tianjin–Hebei region significantly progressed and roughly showed the trend of gradually spreading outwards, with the core exhibiting the high value point of coupling. The coupling degree in most regions represented by the Yangtze River Delta region continued to decrease to the low coupling period.

The overall coupling degree of tourism efficiency and development level in Chinese provinces gradually increased. However, tourism efficiency and development level were in an unrelated and disorderly state from the coupling situation of provincial units. The interaction between them gradually strengthened, or gradually developed in the orderly direction in the check and balance or cooperation, or returned to the game state. The coupling degree of most provincial units tended to rise and fall after reaching benign coupling. The coupling relationship gradually weakened after a certain period of polarization and diffusion effects.

## Conclusion and discussion

### Conclusion

1. The overall distribution characteristics of China's provincial tourism efficiency is high in the east and low in the west during 2000–2020, with evident spatial differences and an overall fluctuating downward trend. Scale efficiency plays a supporting role for comprehensive efficiency, and technical efficiency plays an influencing and constraining role. Tourism development level is at a medium scale level, with high value areas of scale concentrated in the Yangtze River Delta region, the Pearl River Delta region, and the Beijing–Tianjin–Hebei region. The vast majority of provinces exhibit more scope for growth in the tourism development level. The significant increase in technological progress changes is the main factor maintaining positive growth in tourism efficiency. However, scale efficiency is basically unchanged with technological progress, and the overall comprehensive efficiency is fluctuating and in decline, factor allocation is unreasonable, and a low input–output conversion rate leads to poor quality of tourism development. Therefore, tourism development is facing an urgent need for transformation and upgrading, tourism development quality needs to be improved.
2. China's provincial tourism efficiency of the local spatial structure has less overall fluctuation, with the eastern region showing more stability than the western region. The local spatial structure of tourism development level is more stable than tourism efficiency, and its pole-core radiation role is brought into play. For western provinces, it is necessary to fully tap into local resource advantages, improve tourism infrastructure, expand investment in funds, talents, and technology, stimulate tourism development vitality, expand tourism development scale, and optimize tourism investment returns; For the central and eastern provinces, the space for improving tourism efficiency by expanding tourism scale has weakened compared to the western provinces. Therefore, the central and eastern provinces should optimize the allocation of resource elements, accelerate the adjustment of tourism industry structure, increase investment in tourism technology innovation, actively cultivate and introduce high-quality technical and management talents, fully tap into local cultural tourism resources, and leverage technological advantages such as technology and information, Promote innovation in cultural tourism formats in central and eastern provinces, promote high-quality and efficient development of the tourism industry, and provide demonstration for western provinces.
3. China's provincial tourism efficiency local spatial structure is unstable, and changes should be made in the provincial unit. The local spatial structure of the tourism development level is more stable, and it is more difficult to change the relative position of the provincial unit. Tourism efficiency may be enhanced by adjusting tourism development level, but the effect is limited. The level of economic development is an important factor driving the development of tourism and tourism efficiency. Regions with better economic development have more advantages in tourism investment, technology, and scale investment of tourism resources. There are significant differences in tourism resource endowments and economic development foundations among different provinces in China. The development of the tourism industry should focus on differentiation, promote tourism efficiency improvement according to local conditions, and achieve strong sustainable development.
4. The efficiency of tourism, the level of development of the tourism industry, and the coordination between the two have similar characteristics in both time and space. In terms of time, all three increase with the development of time; In terms of space, the three have an overall pattern of high in the southeast and low in the northwest, with some core provinces driving the rise of surrounding provinces. Provinces with higher coordination have higher tourism efficiency and tourism development level, while provinces with lower coordination have lower tourism efficiency and tourism development level. Therefore, there is a positive linear relationship between coordination and tourism efficiency and tourism development level. The higher the degree of coordination, the more efficient, reasonable, and healthy the development of the tourism industry in the region, and it can also indicate that the tourism industry in the region is more developed.

### Discussion

1. Evaluation index selection. Tourism quality measurements can be prioritized by economic development or resource and environmentally friendly orientation. This paper followed the traditional evaluation indexes of tourism efficiency and development level to measure tourism quality from tourism efficiency and development level owing to the limited data of the research unit. It recognized its spatiotemporal evolution and coupling coordination to clarify the regional tourism development path. The selection of different evaluation indicators and evaluation results vary and should be further improved by subsequent research to measure tourism quality and consider the influence of multiple influencing factors on tourism development quality such as environment friendliness, transportation convenience, humanistic veins, and policy conditions to build a tourism quality evaluation system with more general significance that is systematic and comprehensive.
2. Analysis of influencing factors. There are significant spatiotemporal differences between tourism efficiency and tourism development level, based on the comprehensive spatiotemporal evolution analysis of China. The evolution of regional tourism system is a long-term and complex process. Therefore, the spatiotemporal evolution of tourism efficiency and development level is comprehensively influenced by multiple factors. The level of economic development is an important factor driving the development of tourism and tourism efficiency. Regions with better economic development have more advantages in tourism investment, technology, and scale investment of tourism resources. The economic strength of the eastern region results in higher tourism efficiency and development level than the western region. Tourism resource endowment is an important material foundation for the development of tourism in various provinces and regions, and is the main driving force for the spatiotemporal evolution of tourism and tourism efficiency. Tourism attractions are one of the main factors that attract tourists. The improvement of the quality and quantity of tourist attractions helps to improve the scale and efficiency of tourism. Transportation service facilities are a bridge connecting tourism destinations and tourist sources, and are also the main driving force for the evolution of tourism and tourism efficiency. Transportation accessibility has a significant impact on the flow, direction, and velocity of regional tourism flow. Convenient transportation can accelerate and expand the spatial flow of tourists, promote the agglomeration of tourism resources, elements, enterprises, and so on. Transportation hubs and areas along the transportation route have an impact on the temporal and spatial evolution characteristics of regional tourism. Macro policy conditions are the catalyst for the spatiotemporal evolution of regional tourism and tourism efficiency. Policy conditions have driven the improvement of regional transportation conditions, the introduction of technology and talent, and capital investment to optimize the industrial structure and promote the development of tourism and tourism efficiency. Factors such as economic development level, tourism resource endowment, transportation service facilities, and macro policies have an impact on the spatiotemporal evolution of regional tourism development level and efficiency. However, each influencing factor has different driving degrees for the spatiotemporal evolution of tourism development level and efficiency in different time periods and regions. The changes in regional tourism development level and efficiency are the result of the combined action of multiple factors.
3. Coordinated regional development. China's provincial tourism quality shows a clear regional divergence that is high in the east and low in the west based on the three major geographical regions: East, West, and Central. The tourism development foundation of the western region is weak and requires flexibility to learn from advanced technology and experience and optimize the return on tourism investment. The momentum of tourism development in the central region is insufficient to fully exploit their own resource potential and stimulate tourism development vitality. The eastern region progress space is limited to accelerate industrial restructuring and technological innovation. Therefore, it should transfer some of the advantages of resources to the central and western regions. The low efficiency of tourism in the western region is due to lower technological and scale efficiency, and increasing returns to scale. The fundamental reasons lie in the low level of technology and management, poor equipment and facilities, small scale of the tourism industry, limited development of scenic spots, limited number of basic service facilities, and insufficient number and capacity of employees, resulting in the development level of the tourism industry being at a relatively low level nationwide. Therefore, it is recommended to expand the scale of the tourism industry in the western region, increase policy and financial support, expand tourism development channels, improve tourism related supporting facilities, integrate smart tourism elements, and strengthen the business capabilities and service levels of tourism practitioners.
4. Herein, the heterogeneity characteristics were not considered in the analysis of the efficiency and development level, owing to limitations in ability and article length. There are significant differences in industry structure efficiency and development level among different industries such as accommodation, catering and scenic spots; there are also differences among different regions. Another important question to discuss is whether the COVID-19 pandemic has had an impact on the spatio-temporal evolution and coupling coordination of tourism efficiency and development level in China. This significant global event could potentially influence the findings. Therefore, further validation is needed in future research.

## Data Availability

The datasets generated during and/or analysed during the current study are available from the corresponding author upon reasonable request. Figure 1–4 was created using the Free and Open Source Geographic Information System (ArcGIS 10.2) software, QGIS(https://developers.arcgis.com/). The basemaps used to create the maps were downloaded from the National Platform for Common Geospatial Information Services Website: http://bzdt.ch.mnr.gov.cn/download.html.
